# An overview on the RSV-mediated mechanisms in the onset of non-allergic asthma

**DOI:** 10.3389/fped.2022.998296

**Published:** 2022-09-20

**Authors:** Sara Manti, Giovanni Piedimonte

**Affiliations:** ^1^Pediatric Pulmonology Unit, Department of Clinical and Experimental Medicine, University of Catania, Catania, Italy; ^2^Pediatric Unit, Department of Human Pathology of Adult and Childhood Gaetano Barresi, University of Messina, Messina, Italy; ^3^Department of Pediatrics, Biochemistry and Molecular Biology, Tulane University, New Orleans, LA, United States

**Keywords:** respiratory syncytial virus, wheezing, asthma, experimental studies, human studies, immune system, neurogenic inflammation

## Abstract

Respiratory syncytial virus (RSV) infection is recognized as an important risk factor for wheezing and asthma, since it commonly affects babies during lung development. While the role of RSV in the onset of atopic asthma is widely recognized, its impact on the onset of non-atopic asthma, mediated *via* other and independent causal pathways, has long been also suspected, but the association is less clear. Following RSV infection, the release of local pro-inflammatory molecules, the dysfunction of neural pathways, and the compromised epithelial integrity can become chronic and influence airway development, leading to bronchial hyperreactivity and asthma, regardless of atopic status. After a brief review of the RSV structure and its interaction with the immune system and neuronal pathways, this review summarizes the current evidence about the RSV-mediated pathogenic pathways in predisposing and inducing airway dysfunction and non-allergic asthma development.

## Introduction

Respiratory syncytial virus (RSV) is the most common respiratory pathogen in infants and young children worldwide (1). Prospective epidemiologic studies support the association between RSV infection and short-term pulmonary morbidities during infancy, such as lower respiratory tract infections (LRTIs), and long-term pulmonary morbidities during childhood, such as airway hyperresponsiveness, wheezing, and asthma (2–9). Consistent literature findings evidence that RSV-caused asthma is closely related to the atopic constitution since a T helper (Th)2 dominance in immune response has been commonly reported. Studies support an intrisic “Th2-trophic” effects of the RSV as it: (1) stimulates the T cell responses to inhalant allergens, by triggering the local Th2 cytokine at the airway mucosa; (2) promotes eosinophils recruitment at lesional sites in the airway mucosa; (10, 11) and generates a Th2-polarized RSV-specific immunological memory, which, following a RSV reinfection, leads to intense infiltrates of eosinophils and Th2 cells secreting interleukin (IL)-4 in the lung tissue (12).

Whether the role of RSV in the onset of atopic asthma is widely recognized, its impact on the onset of non-atopic asthma, mediated *via* other and independent causal pathways, has long been suspected, but the association is less clear (2–9). The RSV-mediated persistent inflammation and airway hyperreactivity probably result from changes in the local and systemic immune response and alterations of the neural airway pathways that can occur in parallel and/or at different times (13–15). However, all these changes appear reversible, suggesting a transient respiratory dysfunction rather than chronic and irreversible damage, commonly featuring asthma (15). This review aims to summarize the current evidence about the RSV-mediated pathogenic pathways in predisposing and inducing airway dysfunction and non-allergic asthma development.

## RSV structure and host cell interaction

Respiratory syncytial virus is a non-segmented, negative-sense, single-stranded RNA virus belonging to the Paramyxoviridae family whose genome is constituted by ten sequentially arranged genes encoding the following eleven proteins: three transmembrane surface glycoproteins [attachment G protein, fusion (F) protein, the small hydrophobic (SH) protein]; three genomic RNA-associated proteins forming the nucleocapsid [large (L) polymerase, N protein, phospo (P) protein]; two non-structural proteins (NS) (NS1 and NS2); two transcription and replication factors (M2-1, M2-2); and one unglycosylated matrix (M) protein (16, 17). The L, N, P, M-1, and M-2 proteins and the genomic RNA participate in creating the ribonucleoprotein (RNP) complex, and they are required for viral transcription and replication. The soluble form of G protein (Gs) and the NSs (NS1 and NS2) proteins downregulate the antiviral response (18).

While the proteins G and F are crucial for virus attachment and fusion, respectively; the SH protein, a pentameric ion channel, is involved in permeabilizing cell membrane and delaying apoptosis in infected cells (17).

Interestingly, the cytoskeleton plays a supporting role in the infectious cycle of RSV (19). The cytoskeleton is made up of the three following proteins: actin, intermediate filaments, and microtubules. Accounting for 5–10% protein, actin is the most abundant cytoskeletal protein, and it is present both in a globular monomeric form (G-actin) and filamentous form (F-actin) with a different polarity needed for intracellular transport. In response to varying stimuli, actin microfilaments undergo rapid cycles of polymerization/depolymerization to modulate shape changes, cell contraction and migration (20–25). Neural-Wiskott-Aldrich syndrome protein (N-WASP), a member of the WASP family, and ARP2/3 complex have a regulatory role in the actin polymerization (20–25). The role of actin rearrangement in the RSV infection has been confirmed by the evidence that by disrupting actin through cytochalasin D and latrunculin A, a significant decrease in the viral load of RSV occurred (21, 22). Actin is also involved in RSV endocytosis, replication, gene expression, and cell-to-cell spread. Following the virus entry, actin and actin-modulatory proteins facilitate the RSV transcription. Profilin, an actin modulatory protein, is also essential for RSV transcription. *Via* interaction with RSV M protein, actin mediates budding and virion particle transport (23–25). The crucial role of M protein in trafficking the viral particles emerges from the evidence that the absence of M protein leads to the accumulation of RNP complexes in the cytoplasm; thus, the viral filaments cannot be synthesized (26). The M-containing complex anchors the microtubule organizing center (MTOC) and interacts with the mature RNP, creating a M-RNPs which, in turn, complexes with the cytoplasmic tail of G and F to form the budding mature particles which will move to the plasma membrane (27). While the G protein is not required to generate progeny virus, the F protein is crucial, as the M protein associates and sorts into detergent-resistant membranes (DRMs) only when the F protein is present (27). Specifically, the formation of RSV filaments depends on the F-protein cytoplasmic tail (FCT), especially to a phenyalanine (Phe) residue at position 22, as a mutation in Phe22 causes the inability of RSV F to recruit viral proteins and form filaments (28). To avoid any pitfalls in the spreading of the viral particles, RSV modulates the cytoskeletal and actin rearrangement in creating filopodia, finger-like projections constituted by polymerized actin with free-barbed ends, at which can be added additional actin monomers (29). Cdc42, a GTPase, Rac, Phosphoinositide-3-kinase (PI3K), and Rho mediate the filopodia formation (24, 25, 29). The interaction between RhoA and F protein plays a key role in driving the cell-to-cell spread of RSV and creating syncytia, featuring the RSV infection. Higher expression of RhoA and phospho-myosin light chain (pMLC2) were observed following RSV infection, and the incubation with Rho kinase (ROCK), a regulator of actin activity, reversed this effect (30). Phosphoinositide-3-kinase, a family of cellular kinases, acts as a secondary messenger in modulating the phosphorylation of the serine/threonine kinase Akt. Several studies reported that the viral penetration into host cells depends on PI3K signaling activation, and the PI3K activity requires, in turn, the activation of RhO-family GTPases and actin cytoskeleton reorganization. The role of the PI3K signaling results from the evidence that an impaired activity or inhibition of PI3K affects RSV replication (31).

Lastly, the RSV acts by disrupting intermediate filaments to weak the cell, enhance the cell lysis, and favor the release of the viral progeny in the extracellular space (32).

## RSV and immune-pulmonary pathways

### RSV infection and innate immune

Respiratory syncytial virus infection elicits a strong systemic and airway immune response, involving neutrophils, natural killers, dendritic cells (DCs), macrophages and monocytes, eosinophils, T lymphocytes, and inducing the release of several pro-inflammatory cytokines and chemokines (33).

**Figure 1:**
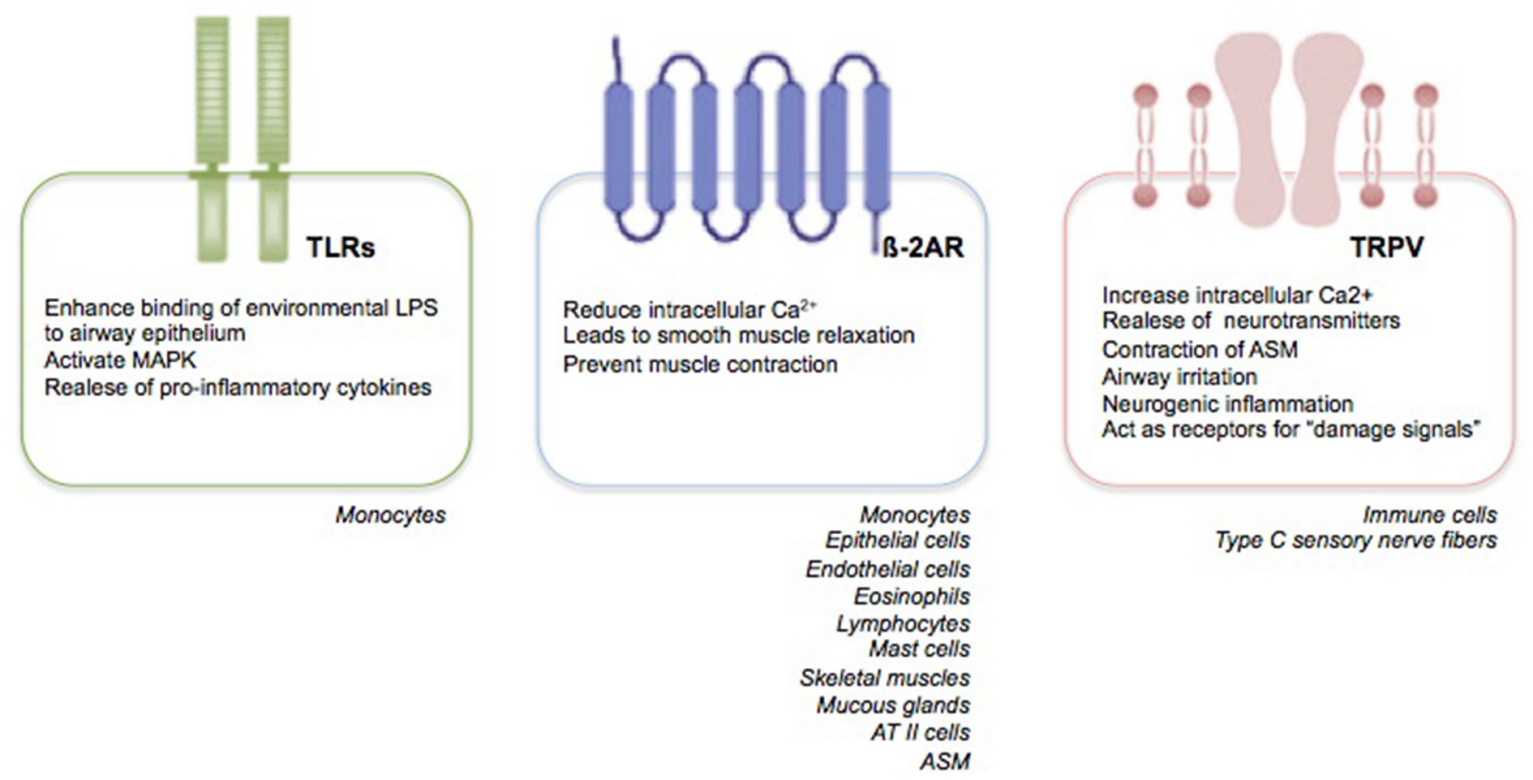
RSV receptors. Expression and function of RSV receptors.

Primarily, RSV targets nasal epithelial cells that release pro-inflammatory mediators and recruit immune cells, such as monocytes-macrophages and DCs (33). Monocytes play two crucial roles: they induce the release of pro-inflammatory cytokines, such as tumor necrosis factor (TNF)-α, IL-1, IL-6, IL-8, IL-10, and IL-18; and promote a Th2-impaired immune response with a parallel decrease in lymphocyte maturation and interferon (IFN)-γ production, and a marked release of IL-4 and IL-13 (34). Recent findings suggest that the human innate lymphoid cells (ILCs), especially the type 2 ILCs (ILC2s), promote also the release of IL-4 and IL-13 with a further enhance of the Th2 immune response (33–35). Moreover, the Toll-like receptors (TLRs) present on the membrane of monocytes, trough the interaction with the RSV F protein, induce an enhanced binding of environmental lipopolysaccharides to airway epithelium, a mitogen-activated protein kinase (MAPK) activation, and a further pro-inflammatory cytokine production [(34, 35), Figure 1].

The RSV G protein affects the innate immune response by binding the motif that resembles the CX3C chemokine fractalkine (Fkn). Mutation or deletion of the CX3C-Fkn motif is associated with higher production of IFNs and TNF-α in response to RSV infection (36).

The SH protein is able to activate the inflammasomes which recruit and activate caspase-1, which, in turn, processes pro-IL-1ß and pro-IL-18 to their active forms (37). Moreover, through inhibition of TNF-α signaling, SH protein delays cellular apoptosis (37).

Through NS1/2, RSV blocks the release and acitivity of type I and III IFNs (38). The crucial role of NS1 and NS2 is confirmed by the evidence that the NS genes' deletion significantly reduced RSV replication in IFN-competent cells but not in IFN-deficient cells (39). Moreover, NS1 and NS2 inhibited the IFN-signaling pathway and the formation of “NS-degradasome,” a complex with a protease/proteasomal activity involved in the suppression of IFN signaling (38).

### RSV infection and adaptive immune

After 4–7 days of infection, the adaptive immune response is activated; however, studies revealed an impairment in T cells response, DCs activity, and T–DC cells interaction, leading to an ineffective memory T cell response to counteract RSV infection (33, 34). Systemic T cell lymphopenia, especially in CD8+ and CD4+, has been reported in patients with early RSV infection compared to convalescence and in uninfected individuals (33, 34). Moreover, an inverse and significant correlation bewteen T cells count and RSV infection severity has also been documented (33, 34).

Given the G and F proteins, RSV elicits the adaptive immune response by inducing the synthesis of RSV-specific antibodies, immunoglobulin (Ig)A and IgG. The RSV-specific IgA are responsible for the defense of the mucosal surfaces, and, moreover, they downregulate the severity of the first infection as well as prevent the reinfection of the upper respiratory tract (40). The RSV-specific IgGs, produced after the first infection, are involved in the viral clearance (41). However, given to the high variability and glycosylation, RSV can change the G protein profile and escape the immune response. By binding the RSV-specific-IgGs, the soluble form of the G protein reduces the serum concentrations of RSV-IgG (42). Moreover, RSV F and G proteins inhibit mitogen-induced T-cell proliferation and decrease T cells functions (39). Lastly, RSV NS1 and NS2 proteins negatively impact the DC maturation into monocyte-derived DCs and affect their ability to interact with T-cell, resulting in a delay in the acquisition of T-cell memory, thus, in a weak adaptive immune response causing susceptibility to reinfection with RSV throughout life (38).

### RSV infection and lung epithelium

From the upper respiratory tract, the virus moves to the lower airways, where it mainly targets ciliated cells and alveolar type II (ATII) cells.

In response to RSV infection, several changes occur at the airway epithelium. It produces cytokines and chemokines that modulate the influx of inflammatory cells into the infected lung tissue. Higher are the cytokines and chemokines levels in the respiratory tract secretions, and more severe is the RSV infection severity. Moreover, RSV, *via* the action of NS2 protein, induces epithelial cell shedding, which, in turn, accelerates the clearance of the virus-infected cells from airway mucosa but contributes to acute obstruction of the distal airways (43). Also, following the infection of basal cells, RSV promotes the IFN-mediated formation of epithelium with a profound loss of ciliated cells (44).

Intraepithelial DCs, alveolar type I (ATI) cells, basal epithelial cells of the bronchial epithelium, and airway smooth muscle (ASM) cells can also be infected by the virus (40). The RSV-infected ciliated cells release pro-inflammatory cytokines such as TNF-α, IL-33, and thymic stromal lymphopoietin (TSLP), which, in turn, promote a Th2-mediated inflammatory response and the recruitment of neutrophils and eosinophils (45). The RSV activates the neutrophils expressing specific activation markers, such as CD11b, CD18, and CD54, and induces the release of neutrophil elastase by neutrophils. Moreover, the neutrophil apoptosis and neutrophil extracellular trap (NET) are active during infection, and the peak of these activities coincides with the maximum in the viral load and clinical severity (46). Similarly, eosinophils are activated by the RSV and the high levels of leukotriene C4, eosinophil-derived neurotoxin (EDN), and eosinophil cationic protein (ECP) detected in the respiratory tract in RSV bronchiolitis support their role during the acute phase of RSV infection (47).

Respiratory syncytial virus may also modulate the human ASM (HASM) function by decreasing the synthesis of cyclic adenosine monophosphate (cAMP) and affecting the ß-2 adrenergic receptor (ß-2AR) functions (48). ß-adrenergic receptors (ß-AR) are transmembrane glycoprotein structures belonging to a major receptor family. They are coupled with guanine nucleotide (GTP) binding proteins (G proteins) and classified in the following three subtypes: ß-1, ß-2, and ß-3 (49). Looking specifically to the ß-2ARs, the latter are coded on chromosome 5 and expressed on epithelial and endothelial cells; eosinophils, lymphocytes, and mast cells; skeletal and uterine muscles; mucous glands, and, predominantly, ATII cells and ASM [(49), Figure 1]. The ß-2AR, existing both in activated and inactivated form, is composed of eight alpha helices; three of which are extracellular, and five intracellular. ß-2 adrenergic receptor is attached to the cellular membrane and transmits the signal intracellularly through heterotrimeric Gs proteins, consisting of alpha, beta, and gamma subunits (49). The catecholamines, such as epinephrine and norepinephrine, are responsible for ß-2ARs stimulation. Additionally, synthetic compounds, known as ß-2AR agonists, and classified in accordance to their duration effects into short-acting, long-acting, and ultra-long-acting drugs, have been tought to stimulate ß-2ARs selectively (50). Following the binding of the agonist ligand to the ß-2AR, the alpha subunit of the Gs protein stimulates the conversion of adenosine triphosphate (ATP) into cAMP, which, in turn, trough the catalytic subunit of protein kinase A enzyme, reduces the intracellular Ca^2+^ concentration, leads to smooth muscle relaxation, and prevents muscle contraction (50). Given their properties, the ß-2AR agonists are part of an effective therapeutic approach to relieve acute airway obstruction, such as during asthma exacerbation; however, they appear less effective when airway obstruction is caused by RSV infection (51). Despite evidence have shown the presence of fully functional ASM also in the early years of life (52, 53) as well as the efficiency of ß-2AR agonists also in newborns and young children (54–56), several clinical trials have failed to demonstrate a clinical benefit of ß-2AR agonists in infants suffering from RSV-mediated bronchiolits (51, 57).

Firstly, Moore et al. (48) investigated the potential influence of RSV on ß-2AR responsiveness by evaluating the isoproterenol (ISO)-cAMP formation, the ß-2AR density, and the Gi expression in HASM cells incubated with RSV. Their findings showed that RSV-infected HASM cells inhibited the ISO-induced cAMP production in a time- and dose-dependent manner and induced a reduction in the ß-2AR density (48). Supporting this evidence, authors showed that RSV could induce an airway insensitivity to ß-2AR-agonists, both directly and indirectly, by inducing heterologous keratinocyte cytokine (KC)/CXCR2-mediated desensitization of epithelial ß-2AR (58, 59). Interestingly, the ß-2AR desensitization occurs in the absence of internalization or degradation of the β2-AR as it results from receptor uncoupling due to phosphorylation by GRK2 (60). More recently, Harford et al. (61) investigated the density and activity of ß-2AR in primary HASM cells derived from pediatric lung tissue with RSV infection compared to non-infected control cells. Their findings showed that RSV induced simultaneously more effects, including a proteasome-mediated cleavage of ß-2AR, a ß-2AR ligand-independent activation of adenylyl-cyclase, and a decrease in cAMP release compared to the control cells, and, lastly, increased intracellular concentrations of Ca2+ resulting in ASM cells contraction (61). Lastly, another explanation for the lack of effectiveness of ß-2AR agonists in infants suffering from RSV infection could be due to the fact that RSV not only induces muscular constriction (bronchospasm) but also impacts the bronchiolar caliber, inducing lymphoid hyperplasia, edema, and mucous plugging, promoting an additional extrinsic compression. Thus, administering ß-2AR agonists did not affect the RSV-mediated airway obstruction (61).

## RSV and neurological pathways

The RSV infection in early life causes airway hyperreactivity and inflammation also attributed to an inappropriate neural control of ASM (62, 63). The airway patency depends on the activity and interaction between adrenergic, cholinergic and non-adrenergic–non-cholinergic (NANC) pathways. The adrenergic system, poorly present in the smooth muscle, releases catecholamines and induces bronchorelaxation. The cholinergic pathway releases acetylcholine and induces bronchoconstriction. The NANC component is constituted by inhibitory (NANCi) and excitatory (NANCe) sub-systems. The first one regulates the relaxation of ASM mediated by neurotransmitter vasoactive intestinal peptide (VIP) and nitric oxide. The NANCe sub-system is constituted by unmyelinated (C-type) sensory nerve fibers, and it causes bronchoconstriction mediated by tachykinins such as neuropeptide substance P, neurokinins A and B (63). Substance P acts by binding NK-1, NK-2, and NK-3, three receptors with a rhodopsin-like structure, also expressed in the immune cells. Among these receptors, the NK-1 has a high affinity for substance P and mediates its pro-inflammatory and immunomodulatory effects, including an increased endothelial permeability; induction of T cells, B lymphocytes, monocytes, and macrophages proliferation and activities; chemotaxis-inducer effects; and degranulation of mast cells. The disruption of the NK-1 protects an immune-mediated lung injury, supporting the role of the P/NK-1 interaction in the RSV infection (59). Moreover, the cells expressing NK-1 receptor also show neural endopeptidase and kininase II, two peptidases that cleave the carboxyl-terminal dipeptide of substance P, thereby, inhibiting its actions. Piedimonte et al. (64, 65) showed the effectiveness of the corticosteroids in preventing the neurogenically-mediated vascular permeability of the airways, as they increase the peptidase activity, and it was completely reversed when both kininase II and neutral endopeptidase were simultaneously inhibited.

The upregulation of nerve growth factor (NGF) and its TrkA and p75NTR receptors has also been reported in a *vivo* model infected with RSV (66). Together with brain-derived neurotrophic factor (BDNF), neurotrophin 3 (NT-3), and neurotrophin 4/5 (NT-4/5), NGF is a neurotrophin (NT) involved in the neuronal development, survival, and function, such as synapse formation and plasticity (63, 67) (Table 1). The NT-mediated effects are the result of their interactions with the p75 neurotrophin receptor (p75NTR) and tropomyosin-related kinase (Trk) family, which may work separately as well as together. The p75NTR, belonging to the TNF receptor superfamily, is a low-affinity receptor for NGF and a receptor for the NTs precursor forms, and it mediates neurite outgrowth, migration, survival, cell cycle arrest, and apoptosis (63, 67). The Trk receptors, which include TrkA, TrkB, and TrkC, interact with all NTs and lead to an activation of several downstream signaling cascades, including PI3K/Akt (protein kinase B a.k.a. PKB) and phospholipase Cγ (PLC) pathways which, in turn, promote the neuronal development, axon and dendrite growth, membrane trafficking, glial differentiation, and interactions with adjacent neurons (67, 68). The crucial role of the NTs and their specific receptors in the RSV-mediated pathogenic mechanisms has been reported in several studies. Increased NGF protein levels and TrkA expression were detected in macrophages and airway epithelial cells in BAL of infants with acute RSV infection requiring ventilatory support (66). In addition, following RSV infection, NGF, by promoting overgrowth of neurites with higher substance P content, favored the sensory fiber responsiveness, acetylcholine and pro-inflammatory peptides release, and long-term remodeling of NANC in the airway (63, 66). Moreover, the NGF over-expression might further affect the ASM tone dysregulation *via* a decrease in catecholamine production, resulting from the adrenal medulla cell differentiation into nerve cells (68–71).

**Table 1:** The neurotrophins: their receptors and neuronal and immune effects.

**Neurotrophins**	**Receptors**	**Neuronal effects**	**Immune effects**
NGF*	Trk*A p75NTR*	Growth and survival of neurons Growth, survival, and differentiation	Eosinophils: Survival and recruitment
BDNF*	TrkB p75NTR	Neuronal plasticity and morphogenesis	Stimulate further NTs release
NT-3*	TrkC p75NTR	Growth of sympathetic axons Increased synaptic strength in nerve-muscle synapses Maturation of proprioceptive and nociceptive neurons Expression of ion channels Expression of neuropeptides Higher excitatory postsynaptic flux in hippocampal areas	Lymphocytes: Differentiation CD80 and CD86 cell surface expression Promote cytokines release DCs*/T-cell interaction Macrophages: Modify macrophage phenotype Inhibit monocyte migration Inhibit antigen presentation Modulate TLR pathways
NT-4/5	TrkBp75NTR	Growth and survival of sensory neurons Survival of dopaminergic and cholinergic neurons, and motoneurons	Mast cells: Survival Promote mast cell tissue infiltration Promote mast cell activation Promote expression of proinflammatory mediators

NGF, nerve growth factor; BDNF, brain-derived neurotrophic factor; NT-3, neurotrophin 3; NT-4/5, neurotrophin 4/5; p75NTR, p75 neurotrophin receptor; Trk, tropomyosin-related kinase; DCs, dendritic cells.

The slow-conducting non-myelinated C-fibers represent up to 75% of vagal bronchopulmonary afferents. They innervate the airways from upper (nose, larynx, trachea) to the lower tract, including the parenchyma and alveolar wall. They express the transient receptor potential (TRP) ion channel family, consisting of 28 ion channels and classified, in accordance with their structure and activation mechanisms, into six subgroups, including: ankyrin (TRPA, 1 channel), canonical (TRPC, 7 channels), melastatin (TRPM, 8 channels), mucolipin (PRTML, 3 channels), polycystin (TRPP, 3 channels), and vanilloid (TRPV, 6 channels) families (72). Specifically, the TRPV family (TRPV1–6) are non-selective cationic ligand-gated channels with high permeability to Ca^2+^. They are commonly expressed by non-neuronal cells, including immune cells and type C sensory nerve fibers of the respiratory tract, and neuronal cells [(73–75), Figure 1]. TRPV family is triggered by exogenous mediators, such as high temperature, osmolarity, exposure to air pollutants, cigarette smoke, allergens and viral agents, capsaicin (CPS); and endogenous stimuli, such as bioactive pro-inflammatory lipids [thromboxanes, prostaglandins E2 (PGE2), leukotrienes, and arachidonic acid derivatives] (73, 74). Following exogenous and endogenous stimuli, TRPVs allow extracellular Ca^2+^ entrance into neuronal cells, which, in turn, leads to the release of neurotransmitters and result in contraction of ASM, contributing to the onset of the airway mechanisms of defense, such as mucociliary clearance, reflex bronchoconstriction, airway irritation, neurogenic inflammation, and cough reflex (76). Moreover, TRPVs act as receptors for “damage signals” able to transfer the signal neuronal fibers to the immune cells, thus, inducing and perpetuating a “pro-inflammatory status,” also attributed to the release of IL-6 and neuropeptides substance P (77).

Recently, the TRPV family has gained great interest from researchers, which focused their attention, especially on the functions of TRPV1 and TRPV4, mainly expressed in the respiratory tract. Because TRPV1 channels are commonly co-localized with sensory neuropeptides, including calcitonin gene-related peptide (CGRP) and tachykinins, the TRPV1 activation causes a “neurogenic inflammatory reaction” featured by bronchoconstriction, inflammatory cell chemotaxis, and airway mucosal oedema (78). TRPV4 is expressed in mammalian tissues, including lung (human bronchial epithelial cells and alveolar wall), brain, sensory neurons, sympathetic nerves, salivary gland, sweat glands, inner ear, heart, kidney, intestine, skin, endothelium, and fat tissue (79). The TRPV4 activation induces both the activation of K+ and Ca^2+^ channels, resulting in a further ASM contraction (80). Taking into account their functions, it well appears how a prolonged and intense stimulation of TRPV1 and TRPV4 plays a crucial role in the pathogenesis of some airway diseases, such as chronic cough and asthma, as well as viral-mediated airway damage, since TRPV1 and TRPV4 are both involved into host–pathogen contacts including the binding, entry and replication of the viruses (81, 82). In this regard, in a *vivo* model, authors firstly reported that the RSV induced a neurogenic inflammation mediated by TRPV1 and capsaicin, resulting from the upregulation of NK-1 both in the airway epithelium and vascular epithelium (83). Later, the same authors reported that, during the RSV infection, the stimulation of the TRPV1-expressing sensory nerves was also involved in the overexpression of NK-1 receptors in CD4+ T cells and also showed chemotactic effects on this subset of lymphocytes (84). Furthermore, inoculation of *in vivo* model with RSV, induced over-expression of NGF, phenotypic switch in tachykininergic innervation of the airways, and a long-lasting airway inflammation (67, 85, 86). In line with these findings, in a *vitro* model, Omar et al. (81) reported an up-regulation in TRPV1 expression after 12 h post-RSV infection. Interestingly, this effect was independent of replicating virus as the virus-induced soluble factors were sufficient to increase channel expression. Moreover, the inhibition of RSV infection by using capsazepine induced a down-regulation of TRPV1, suggesting that these receptors have key role in virus-induced airway damage (81). Similarly, Jing et al. reported a down-regulation in the TRPV1 signaling pathway and in airway inflammation and mucus hypersecretion when qingfei oral liquid was administered to RSV-infected asthmatic mice models (87). More recently, this evidence was confirmed in human bronchial epithelium from children with asthma both at baseline and after RSV infection (88). Specifically, children with asthma were intrinsically reporting higher basal TRPV1 protein expression when compared to children without asthma; moreover, a further increase in TRPV1 expression was also noted in asthmatic children during RSV infection since the virus promoted higher intracellular Ca^2+^ levels as well as NGF overexpression (88). In addition to the asthma status, the patient's age is also a factor affecting the TRVP1 expression during RSV infection (89). By comparing the TRPV1-mediated Ca^2+^ changes in human bronchial epithelial cells from children and adults with and without asthma, at baseline and after RSV infection, authors noted that TRPV1 expression, localization, and activity were higher in asthmatic children but not in adults, supporting the evidence that RSV entry and/or replication is more efficient in the bronchial epithelium from children but not in adults, a population in which RSV did not affect the TRPV1 function regardless of the asthma status (89).

## RSV and non-allergic asthma: *In vivo* and human models

Since it commonly affects babies during lung development, the RSV is widely recognized as an important risk factor for wheezing and asthma. Although the role of RSV in the onset of atopic asthma is widely recognized, its impact on the onset of non-atopic asthma, mediated *via* other and independent causal pathways, has long been also suspected but the association is less clear. Following RSV infection, the release of local proinflammatory molecules, the dysfunction of neural pathways, and the compromised epithelial integrity can become chronic and influence airway development, leading to bronchial hyperreactivity and asthma.

### *In vivo* models

Although human studies are essential to assess or refute data from experimental models, many of RSV's behaviors have been reproduced and replicated in the murine models, as they are highly susceptible to RSV infection, permissive to viral replication, and are strictly reflecting the virus and specific T and B cells interactions. In infected animals, RSV infection causes pulmonary damage similar to that observed in humans, featured by degeneration of nasal epithelial mucosa, peribronchiolitis, interstitial pneumonitis, and perivasculitis (10, 11). Accordingly, several studies in animal model systems, firstly, highlighted the key role of Th2-polarized response in the immunopathogenesis of RSV-induced airway inflammation (10, 11). Later, an “asthma-promoting” effect of the RSV infection, regardless of atopic status, has also been postulated (90). Studies conducted on Balb/C model showed that a population of IFN-γ-secreting CD8+ T cells, potentially attenuating the pathogenic Th2 host response to the RSV G-protein, was involved in the RSV-induced airways inflammation (91).

Authors postulated that the age at first infection determined the type of cytokine production and, consequently, the disease patterns during reinfection (12). To investigate the rechallenge, mice were infected at 1 day or 4 or 8 weeks of age and reinfected at 12 weeks. While neonatal priming produced a more severe inflammatory cell recruitment, such as Th2 and eosinophils, delayed priming led to an increased IFN-γ production and a less severe disease in later life. These results showed the crucial importance of the age at the first infection in determining the outcome of reinfection and suggested that the environment of the neonatal lung is a major determinant of cytokine production and disease patterns in later life (12). *In vitro* studies have also shown that IFN-γ activates eosinophils, prolongs their survival and promotes the synthesis of leukotrienes from these cells. Considering this evidence, Wedde-Beer et al. postulated that leukotrienes, originating from the interaction between P-containing nerves and mast cells, may be important mediators of RSV-induced airway inflammation (92). Accordingly, rats were inoculated at 2 or 12 weeks of age with RSV or virus-free medium and treated with montelukast or its vehicle starting 1 day before inoculation. The authors reported greater microvascular permeability in the intrapulmonary airways of RSV-infected rats not receiving montelukast treatment compared to the control group. Moreover, a significant increase in 5-lipoxygenase-encoding mRNA and cysteinyl leukotrienes levels and mast cells was detected in the lung tissues of RSV-infected rats, suggesting that the increase in vascular permeability might be promoted by mast cells-derived leukotrienes (92).

The potential effect of the RSV-induced upregulation of NGF must also be considered as a pathogenic mechanism in the onset of non-atopic asthma. Because NGF is released from airway epithelial cells, it increases the release of substance P and other tachykinins from adult sensory neurons and induces sensory hyperinnervation in the airways of transgenic mice. Substance P, in turn, activates mast cells releasing leukotrienes, which further sensitize C-type neurons to release neurotransmitters, thereby reactivating the mast cells and creating a vicious circle contributing to an exaggerated inflammation of the lower respiratory tract (92). In a mouse model, the expression of NGF, trkA, and p75 declined with age, but the RSV increased their levels both in weanling and adult rats. Confirming the role of the NGF and its receptors as major determinants of neurogenic inflammation in RSV infection, authors also reported that exogenous NGF upregulated NK1 receptor expression in the lungs and, on the contrary, the anti-NGF antibody inhibited NK1 receptor and, thereby, the neurogenic inflammation in RSV-infected lungs (86). The evidence that NGF expression was inversely correlated with the age, supported the hypothesis that this neurotrophin is critical for the neuronal plasticity; thereby, variations in its expression can result in permanent changes in sensorineural lung pathways further contributing in airway hyperresponsiveness and asthma susceptibility (93). These findings were confirmed by Piedimonte et al. (79) that showed an increase in NGF and neurotrophin receptor expression in early life during RSV infection and a RSV-related abnormal remodeling of neuronal networks in the respiratory tract, resulting in bronchial hyperreactivity and airway obstruction. Interestingly, the neurogenic inflammation and the bronchial hyperreactivity were long lasting in mice up to 60 days after intranasal inoculation of RSV (94). Confirming these findings, animal data revealed that the RSV-induced airway hyperreactivity was long-lasting for weeks after the inoculation of the virus (95–97). This protracted inflammatory response may be due to the persistence of viral genomic in mouse lung tissue, which has been demonstrated to last for at least 67 days following RSV inoculation (95–98).

New data support the Th17 cell differentiation during RSV infection and Treg airway accumulation during RSV clearance (99). In a mice model, authors found that RSV infection, through activation Notch-1/DLL3, increased the mRNA expression of IL-17A and IL-17A/Foxp3 and Treg levels in the hilar lymph nodes and mesenteric lymph nodes. In contrast, the mRNA expression of IL-4 and other Th2 cytokines was unchanged (100, 101).

Recently, it has been reported that RSV infection contributes to the onset of asthma in later life by inducing High Mobility Group Box-1 (HMGB1), as a result of necroptosis, a programmed cell death of airway epithelial cells (102, 103). The inhibition of necroptosis decreased the severity of bronchiolitis by reducing viral load, prevented the airway epithelial cells remodeling, and the asthma development in later life (102, 103).

In addition to the immune dysfunction and neurogenic inflammation, non-atopic asthma would be the result of an impaired relationship between epithelium and mesenchymal structures. An exaggerated release and responsiveness to trophic factors and a subsequent abnormal growth of smooth muscle, nerves, and blood vessels are considered crucial events in remodeling the airways. The barrier dysfunction could enhance the sampling of luminal antigens by intraepithelial DCs, and facilitate translocation of inhaled particles, allergens, bacterial and viral pathogens through the lung, resulting in an inappropriate immune response and airway inflammation (104, 105).

The integrity of the airway epithelial barrier is regulated by several mechanisms, including the assembly of the epithelial apical junctional complex (AJC), which are composed of apically located tight junctions (TJs) and underlying adherens junctions (AJs), also containing adhesion, scaffolding, signaling, and cytoskeletal proteins (104). The TJs limit the passage of ions and uncharged solutes thanks to their adhesive properties. Three major types of transmembrane proteins are described: (1) members of the claudin family, (2) the TJ-associated MARVEL proteins (TAMP) family, which includes occludin, tricellulin, and Marvel D3, and (3) immunoglobulin-like proteins, which include junctional adhesion molecule A (JAM-A) and coxsackievirus and adenovirus receptor (CAR) proteins (104, 105). The AJs are involved in starting and maintaining of epithelial cell–cell contacts, and enabling TJ assembly. The E-cadherin and nectins are the main transmembrane adhesion proteins involved in forming epithelial AJs, cell–cell adhesion, cell signaling, proliferation, and differentiation. The cytoplasmic side of TJs is organized by multifunctional scaffolding proteins of the zonula occludens (ZO) family, whereas β-catenin, α-catenin, and p120 catenin form a complex with the cytoplasmic domain of E-cadherin (106). C57BL/6 mice, intranasally inoculated with RSV, showed a significant peribronchial inflammation compared with non-infected controls and UV-inactivated RSV-inoculated animals. Moreover, RSV infection increased the permeability of the airway epithelial barrier, decreased the expression of several TJ proteins, and prevented the accumulation of cleaved extracellular fragments of E-cadherin in BAL and tracheal epithelial cells (107). Another study found that RSV infection led to decreased mRNA expression of claudin-1 and occludin in lung samples of wild-type BALB/c mice (108). These studies supported the evidence that RSV induces a AJCs disorganization and dysfunction, and it was likely a result of cortical F-actin cytoskeletal remodeling, which is, in turn, is regulated by Protein kinase D (PKD)-mediated phosphorylation of cortactin, an actin-binding protein that regulates F-actin dynamics between polymerization and depolymerization steps during plasma membrane remodeling (109).

Globally, an abnormal remodeling of the airways substructures, especially involving the mucosal neural network, occurs when the airways are infected during critical developmental windows in early life. However, because these changes seem to be the result of a transient derangement in airway development, these modifications would return to baseline condition when the virus is cleaved, thereby, justifying the evidence that some children with previous RSV infection do not develop asthma. On the other hand, RSV-associated long-term consequences have been reported in neonatal mice, such as persistent mucus production, subepithelial fibrosis, and increased collagen deposition, contributing to promoting and maintaining airway remodeling also in later life (109–111). Moreover, 30 days after inoculation, neither evidence of active RSV infection nor immunostaining and no viral nucleic sequences were detected in a mouse model. In contrast, the substance P in the lung and the capsaicin-induced plasma extravasation were significantly higher in the infected mice compared with pathogen-free rat, supporting the evidence that the sensory innervation of airways remain susceptible to the proinflammatory effects even if RSV infection is disappeared (112). In conclusion, RSV seems to exert a dual influence: in short-term postsynaptic manner, deriving from up-regulation of the substance P, and in long-term presynaptic manner, by remodeling sensory innervation (112).

### In humans

Similarly to other viruses, RSV shows a close relantionship with the development of wheeze and asthma in later life, regardless atopic status (2, 113). Several birth cohort studies reported that one-third of children with RSV developed recurrent wheezing and asthma, but not allergic sensitization (90, 114, 115). In line with these findings, the Tucson respiratory study reported that children with a previous RSV infection were commonly experiencing wheezing and lower forced expiratory volume at first second (FEV1) by the age 6 years (90). Moreover, the risk decreased significantly with the age and it was not significant by age 13 (90). There was not any significant relationship with atopic status development; as sensitization did not appear as a risk factor for infants suffering from RSV-caused wheezing (90, 116). By enrolling 95,310 children by Carroll et al. (117) and Wu et al. (9), the Tennessee Asthma Bronchiolitis Study (TABS) suggested a causal relationship between severe RSV infection and the onset of asthma by age 5.5 years. They also reported that during the winter months was recorded the greatest number of bronchiolitis-related hospitalizations. Moreover, the authors observed that children born 4 months prior to the annual peak of bronchiolitis-related hospitalizations were 29% more likely to develop asthma compared with infants born 1 year from this time point. This trend was similar troughout the 5-year study although the peak of bronchiolitis-related hospitalizations shifted by up to 6 weeks. Furthermore, if on one side the authors did not state if children were infected with a specific virus, including RSV, on the other hand, up to 70% of severe bronchiolitis were due to RSV infection (118). In accordance with several longitudinal studies, these changes appears to be transient rather than persisting over the time. Pullan et al. (119) showed that children younger than 1 year of age and with severe RSV infections experienced wheezing primarily during the first 4 years of life, while, by age 10 years, no significant difference in incidence are reported compared to the control group. Similar results were observed in the Tucson Children's Respiratory Study of 1,246 children as children, who experienced a severe RSV infection by up to 3 years of life, were at a significantly increased risk of wheezing at ages 6 and 11 years. However, by age 13 years no significant differences were recorded compared to the control group (120). These data were not confirmed by the longitudinal study by Sigurs et al. (121). Authors described a significant increase in asthma by age 13 years; probably, the differential findings reported in the studies above reported are due to several factors involved in the RSV-asthma relationship, including genetic variability among investigated cohorts as well as the difference in pathogenicity of circulating RSV strains (121).

In two controlled, randomized, double-blinded trials performed in preterm infants receiving palivizumab to prevent RSV bronchiolitis, authors reported that the palivizumab administration was protective against recurrent wheeze up to 3 year of life; supporting the evidence that an early-life RSV bronchiolitis can have a continuing causal impact beyond infant infection (122, 123). Confirming these findings, authors demonstrated that RSV prophylaxis in non-atopic children decreased the risk of recurrent wheezing up to 80% and, interestingly, it did not have effects on infants with a positive family history for atopy (124). Later, a Finnish study reported a significant association between RSV infection and self-reported asthma in adolescents aged from 15 to 18 years (125). In a 7 year follow-up study enrolling 127 steroid-naive children, authors reported that the first severe RSV/rhinovirus–negative wheezing episode was a risk factor for developing non-allergic asthma, together with the age of first episode of wheezing <12 months, and exposure to tobacco smoking (113). Despite this increasing body of evidence supporting a relationship between RSV infection and non-atopic asthma, it remains unclear whether the virus contributes really to the onset of asthma or is simple trigger in subjects with asthma susceptibility (126). Authors hypothesized that probably the association between RSV infection and asthma could be also due to a shared genetic predisposition, as reported in a large population of 8,280 twin pairs in Denmark (126). Furthermore, genetic polymorphisms in the gene encoding IkBa, a negative regulator of NF-kB, have been recently associated with RSV and asthma (127). The gain-of-function polymorphisms in the promoter region of IL-8, a chemokine secreted by epithelial cells and macrophages, lead to an increase in RSV infection severity and in developing wheezing and asthma (128–130). Polymorphisms in other chemokines, such CX3CL1, CX3CR1, and CCL5, seem further predispose to asthma onset (131, 132). Mainly, CX3CR1, expressed in human airway epithelial, binds RSV G protein and mediates the viral entry in the host cells; thus, CX3CR1 mutations and/or complete or partial deletion of RSV G protein results in a less efficient viral entry and a decreased virus replication (133).

Gain-of-function polymorphisms in IL-4 and IL-13 cytokine genes as well as deficit in IFN-γ expression have been also associated with developing wheezing during at the age 6 years (134, 135).

Several mechanisms causing RSV-mediated long-term pulmonary effects have also been investigated. The effects of maternal RSV infection on postnatal offspring immunity and neurotrophins release, ASM contractility, and development of wheezing and asthma are still under debate. However, the recent discovery that viral antigens can impact postnatal immunity *in vivo* model and negatively affect the perinatal period in humans showed the possibility that a challenge in immunity can ocurr after postnatal virus (136, 137). *In utero* exposure to RSV caused a chronic airway dysfunction by influencing cell-mediated host immunity, local NGF expression, neurotransmitters release and neuronal hyperreactivity, and ASM contractility during RSV-mediated LRTIs in early-life (66, 138–140). Neurotrophin levels and immunoreactivity for neurotrophin-receptor were found to be strongly increased in the BAL fluid of mechanically ventilated infants with severe RSV infection (66). Children with severe RSV-bronchiolitis are reporting an increased bronchial responsiveness and altered epithelial immune response to the viral agents compared with children without bronchiolitis (141). It seems that in subjects with chronic obstructive pulmonary disease, the RSV can persist contributing to the pathogenesis of stable disease (142). Smyth et al. (143) studied the behavior of mediators of lymphocyte activity [IL-4, CD25, and soluble intercellular adhesion molecule 1 (sICAM-1)] in 94 children affected by RSV-mediated bronchiolitis. Authors reported that the serum CD25 levels were elevated during acute infection and they remained raised during convalescence up to 150 days after infection, also in absence of acute infection (143). This finding was in contrast to the rapid decline in serum CD25 levels recorded in children after acute infection mediated by other viral agents, such as measles or dengue fever (143). Thereby, RSV could mediate a persistent inflammatory response in the airway that continues for longer than is generally believed. Similarly, Pala et al. (144) compared the production of cytokines of children 7 years after acute RSV bronchiolitis to healthy children. The post-bronchiolitic children showed a significant response in cells producing IL-4, suggesting the role of this cytokine in the RSV-mediated long-term airway effects (144). Moreover, since RSV induced a strong release of IL-10, IL-11, and prostaglandin E_2_ (PGE_2_), molecules well-known for their immunosuppressive effects, authors hypothesized that these mediators might be responsible for the delayed protective RSV specific immune response, further contributing to RSV-mediated long-term damage in the airway (145). More recently, Bertrand et al. (146) reported that the cytokine (IL-3, IL-4, IL-10 and IL-13) and chemokine (IL-1β, IL-6, TNF-β, MCP-1/CCL2, MIP-1α/CCL3, and IL-8/CXCL8) levels in the BAL and nasopharyngeal aspirates of children with RSV-mediated bronchiolitis were significantly higher compared to control group, and, moreover, a direct correlation between IL-3 and IL-12p40 levels and development of wheezing later in life was also observed.

Other possible mechanism might include the “hitchhike effect” yet described in patients suffering from chronic bacterial colonization. Specifically, it seems that the neutrophilic airway inflammation following a chronic colonization could contribute to development asthma in the pediatric population (147).

**Figure 2:**
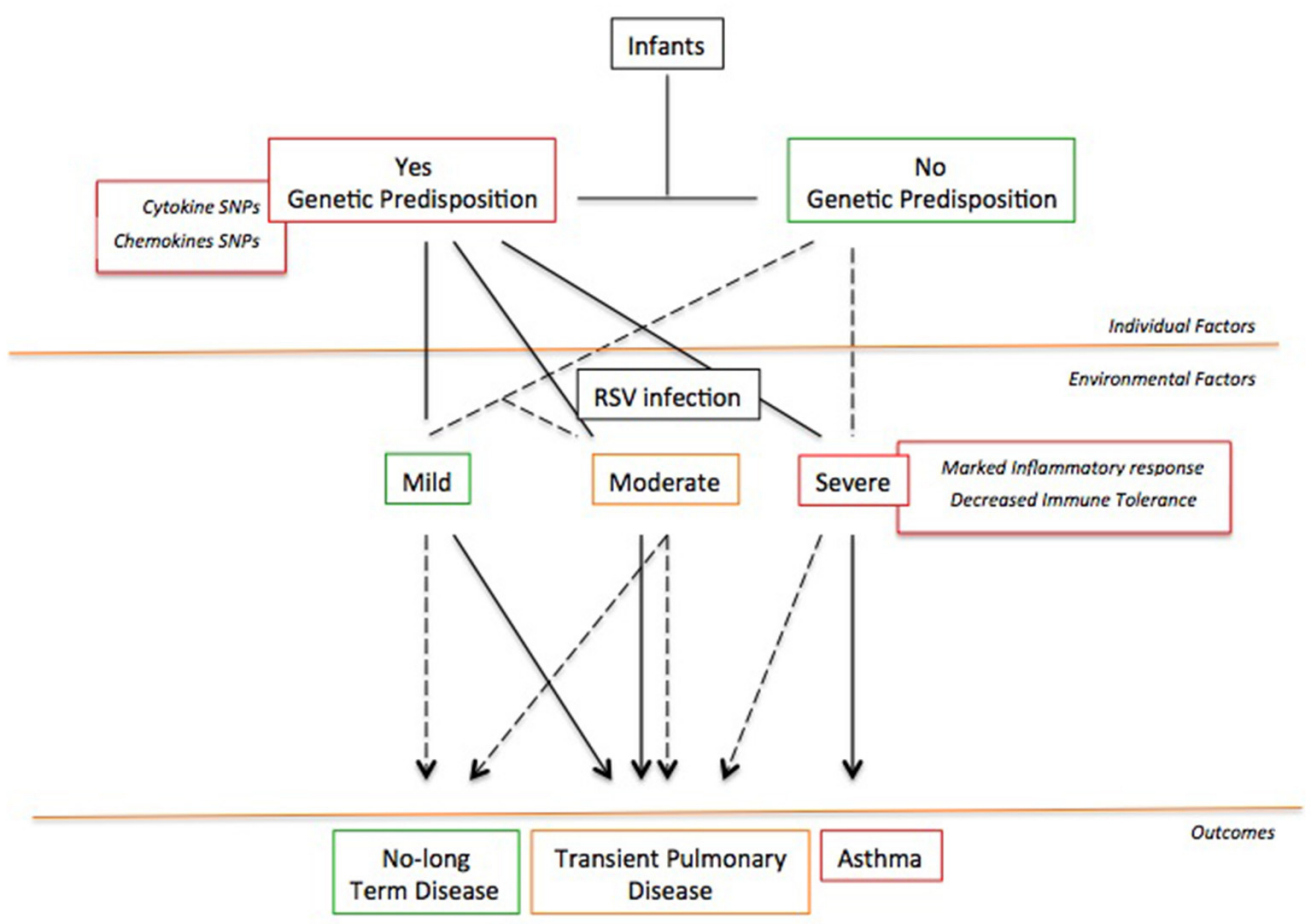
The “Two-hit” hypothesis. The co-existence of at least two or more risk factors favors the asthma development following a severe RSV infection.

Probably, the development of RSV-induced asthma requires a “two-hit” model (148) featured by the co-existence of at least two among individual (genetic, immune response in the lung environment), developmental (lung remodeling), and environmental (exposure to inhalants) factors (Figure 2). Whether only one factor is present, the patient will not develop chronic airway inflammation and asthma; conversely, whether two factors are coexisting, the patient will report long-term symptoms and/or asthma (148). This might explain why not all children with severe RSV bronchiolitis develop asthma as well as why other children with early wheezing show resolution of their illness by their adolescence (148).

## Future prospectives

Compared with other environmental factors, RSV infection remains the main cause of LRTIs in infants, and wheezing and asthma during childhood. Despite several experimental and human studies have demonstrated a close relationship between RSV infection and the subsequent lon-term airway effects, to date, the exact mecahnism underlying the RSV infection and asthma development remains to be elucidated. Respiratory syncytial virus shows an intricate relationship with the local and systemic immune response of the host. Consistent literature findings evidence that RSV-caused asthma is closely related to atopic constitution, since a Th2 dominance in immune response has been commonly reported. Additionally, other important cellular mediators of RSV-mediated inflammation and immune responses are endothelial cells, lymphocytes, macrophages, and mast cells. Moreover, despite its simple structure, RSV shows a complex relationship also with neuronal pathway of the airways. Respiratory syncytial virus makes the airways abnormally susceptible to the RSV-caused proinflammatory effects by upregulating NK-1 receptor gene expression and, thereby, increasing the synthesis of the substance P and the density of its receptors on target immune cells, including lymphocytes, macrophages, mast cells, and endothelial cells. Thus, RSV can establish crucial interactions between neuronal airway system and immune response that result in long-term airway dysfunction, predisposing to the onset and maintaninance of chronic persistent airway hyperreactivity and inflammation (149–151). Additionally, the RSV-immune system-neuronal pathway interactions are influenced not only by the viral factors but also by host factors, such as genetic susceptibility, that modify the efficiency of the response to the virus, viral replication, and virus-mediated injury to the airways. All this may justify the differences in severity infection, damage extention, and duration and magnitude of the RSV effects in later life among the pediatric population. Thereby, due to the pathophysiologic mechanisms involved in the RSV infection, it is likely that future treatment strategies should be focused on modulating the interactions between the virus, host immune response and neuronal pathways. Currently, RSV-mediated treatment is limited to supportive care; and no vaccine is licensed to prevent RSV infection. The only prevention strategy available is palivizumab, which is indicated only in a cluster of preterm newborns or those with comorbidities. On the other hand, vaccine development has encountered several challenges, such as the immaturity of the immune response in infants; thus, most newborns remain unprotected against RSV. It appears that the two feasible strategies for protecting all infants against this virus are maternal immunization and immunization of infants with long-acting monoclonal antibodies (mAbs). The latter seems to provide consistent protection against RSV for at least 5 months, covering the duration of the RSV season, offering great flexibility in the timing of administration, and regardless of the gestational age, presence of comorbidities, and maturity of the immune system. Accordingly, using long-acting mAbs appears to be the only available strategy for protecting all newborns entering their first RSV. Moreover, using long-acting mAbs could also postpone the risk of RSV-mediated bronchiolitis throughout life. As reported above, the age at the first infection represents one of the risk factors for developing non-allergic asthma. Earlier the newborn meets the virus, more severe it will be the airway damage. The *early* RSV-related abnormal remodeling of neuronal networks in the respiratory tract will lead to bronchial hyperreactivity and airway obstruction; thus, the baby will experience *early* wheezing during the first 4 years of life (12, 85, 113). On the contrary, if the child meets the virus *late*, it is reasonable to hypothesize that the RSV-mediated damage to the airways will be less severe. The baby will develop a more mature immune response and will have an advanced lung maturity; therefore, the onset of wheezing will be *late*, or it will not occur (12, 85, 113).

Lastly, a global understanding of the principal mediators and the risk factors for onset of wheezing and for its progression to asthma is critical for prevention strategies after an initial RSV infection as well as for the development of effective therapeutic strategies for viral-induced wheezing/asthma, especially in the pediatric population.

## Author contributions

SM and GP reviewed the literature on the subject, drafted the final version of the manuscript, finally approved the version to be published, and agreed to be accountable for all aspects of the work in ensuring that questions related to the accuracy or integrity of any part of the work are appropriately investigated and resolved. Both authors made substantial contribution to the conception of the work.

## Conflict of interest

The authors declare that the research was conducted in the absence of any commercial or financial relationships that could be construed as a potential conflict of interest.

## Publisher's note

All claims expressed in this article are solely those of the authors and do not necessarily represent those of their affiliated organizations, or those of the publisher, the editors and the reviewers. Any product that may be evaluated in this article, or claim that may be made by its manufacturer, is not guaranteed or endorsed by the publisher.
